# ACTIVITY GOAL SETTING ON CHRONIC LOWER BACK PAIN FOR OLDER VETERANS RECEIVING CHIROPRACTIC CARE

**DOI:** 10.1093/geroni/igac059.2878

**Published:** 2022-12-20

**Authors:** Casey Rogers, Madeleine Hackney, Lisa Zubkoff, Katharina Echt

**Affiliations:** Birmingham/Atlanta VA GRECC, Birmingham, Alabama, United States; Emory School of Medicine, Atlanta, Georgia, United States; GRECC, Birmingham, Alabama, United States; Birmingham/Atlanta GRECC, Birmingham, Alabama, United States

## Abstract

There is currently no literature addressing the impact that chiropractic services have on older adults achieving individualized specific goals throughout a course of care for chronic low back pain. This study aims to explore the impact of setting a self-determined, “what matters most” activity/goal of rehabilitation care with relevant activities as part of standard chiropractic care on the self-rated pain and disability of older Veterans. Participants were randomized into two groups. The first, an experimental group where participants identified a goal and received standard chiropractic care. The second, a control group that received standard chiropractic care only. Participants underwent six sessions of care. Outcome assessment tools were utilized at pre- and post-treatment for both groups as primary measures and an individualized goal setting measurement tool was utilized for those randomized to the experimental group. After treatment, all participants had self-reported improvement in their condition and all participants assigned to a “goal setting group” achieved their desired goal. Despite some outcome measures remaining unchanged, this self-reported improvement may be important in the future of chiropractic services for the older Veteran population suffering with chronic low back pain in achieving self-determined goals of importance.

